# Effect of the characteristic town policy on sewage treatment in mountainous areas: Evidence from Chongqing

**DOI:** 10.1016/j.heliyon.2023.e22830

**Published:** 2023-11-27

**Authors:** Chao Zhou, Qin Wang

**Affiliations:** Sichuan International Studies University, Chongqing, 400031, China

**Keywords:** China, Characteristic town policy, Sewage treatment, Differences-in-differences

## Abstract

The report to the 20th National Congress of the Communist Party of China highlighted the necessity of advancing a new type of urbanization with people at the core. Characteristic towns as an essential link between new urbanization and rural revitalization attach great importance to creating a beautiful environment. Sewage treatment is an indispensable foundation for a town's high-quality economic and social development; however, it may be hard to achieve through urban construction. Therefore, using data from 584 organic towns in Chongqing, a typical mountainous city in western China, this study empirically analyzes the effect of the characteristic town policy in China on mountainous sewage treatment and its mechanisms from 2014 to 2020. The results indicate that (1) the characteristic town policy helps enhance sewage treatment in small towns, which remains valid after a series of robustness tests. (2) Investment and human resource effects are mediators for the characteristic town policy to boost sewage treatment in small towns. Further analyses reveal that the boosting effect of the characteristic town policy on small-town sewage treatment is more significant for small towns with higher economic development levels suffering ecological and environmental pressures. These findings provide a basis for a broad recognition of the effect of the characteristic town policy and its impact. They also theoretically enrich the awareness of the Chinese government's urbanization policy concerning the economy and society.

## 1 Introduction

**Fig. 1:**
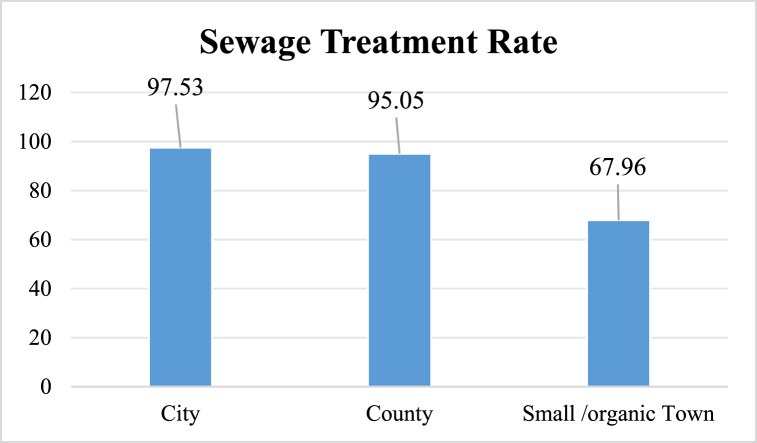
Sewage treatment rates of Chinese cities, counties, and small towns in 2020.

In 2020, sewage treatment rates of cities and counties in China exceeded 97 % and 95 %, respectively (Fig. 1.). However, the sewage treatment rates of small towns still need improvement. During the same period, approximately 35 % of organic towns and 65 % of townships did not treat domestic sewage.

Sewage treatment is an integral base for urban development, a necessary guarantee for achieving green development and improving the quality of the healthy lives of residents; however, it is a problematic point for urban construction. In contrast to cities, small towns have notable weaknesses in many aspects, such as construction costs, operation and maintenance, business models, and capital investment in sewage treatment facilities [1,2]. Small towns with large rural areas bear a heavy responsibility for environmental protection as the “tails” of cities and “heads” of rural areas. Due to the complexity of geographic forms and unsound drainage systems, collecting massive amounts of domestic sewage is often challenging. Most sewage is discharged without treatment, which is hazardous and hard to control. Driven by the rural revitalization policy, central and local governments have devoted substantial resources to the rural living environment and provided support in financing policies. However, sewage treatment still faces many challenges such as weak foundation, heavy environmental pressure, and deficient social capital due to the adverse impact of external epidemics and insufficient internal innate endowments [3,4].

Unlike cities, small towns rank low in China's urban system, with production factors such as a large population and capital being siphoned off by higher-ranking cities; hence, various small towns in China are experiencing severe development bottlenecks. To respond to the growing urban-rural gap in China's urbanization, the hollowing out of township industries, and the increasing pressure on the ecological environment, the Ministry of Housing and Urban-Rural Development, the National Development and Reform Commission, and the Ministry of Finance jointly issued the *Notice on Cultivation of Characteristic Towns* in July 2016 for the nationwide cultivation of “characteristic towns”. They announced the list of the first batch of characteristic towns in October of the same year. Hence, building characteristic towns has become China's national strategy. Documents such as the *Notice on Cultivation of Characteristic Towns* emphasize environmental construction while stressing industrial development, which requires beautiful town patterns in harmony with the surrounding natural environment. Since 2017, provinces and cities have announced provincial-level characteristic towns and have provided financial support. For example, in Chongqing, a municipality directly under the central government in midwestern China, municipal departments invested 350 million yuan annually to construct characteristic small towns. They demanded that districts and counties provided 1:1 matching funds.

The characteristic town policy is critical for the Chinese government to promote rural revitalization strategies and implement new development concepts. However, existing studies on the characteristic town policy have mainly assessed its economic effects, with few studies evaluating the social consequences of the policy and no literature examining the impact of the characteristic town policy on sewage treatment. Numerous studies have examined the effect of urban construction on the ecological environment; however, consistent conclusions have yet to be reached. Moreover, previous studies have focused on cities, with insufficient concern for small towns being the political, economic, and cultural centers in rural areas. In addition, cities have also dominated sewage treatment studies, with few analyses of mountainous towns, where treatment is challenging, especially in the upper reaches of the Yangtze River, where ecological and environmental pressures are considerable.

To fill this gap in research, this study, with 584 organic towns of Chongqing as the research objective, focuses on the sewage treatment effect of the characteristic town policy, exploring whether their introduction and subsequent significant financial investment improve the sewage treatment of mountainous small towns with substantial ecological pressure and prominent sewage treatment difficulties. Moreover, based on the theory of urban-rural integration and the theory of equalization of essential public services, the action mechanism of the characteristic town policy in mountainous sewage treatment is clarified. A model design and baseline regression analysis are proposed based on practicality and robustness. The impact mechanisms and heterogeneity are further analyzed. With such analysis, this paper expects to make strong explorations to promote the in-depth implementation of the characteristic town policy in China in the context of rural revitalization, improve the efficiency of sewage treatment in small towns and alleviate their ecological environmental pressure, so as to ultimately promote the high-quality economic development.

This study's marginal contribution has two primary aspects. First, it examines the impact of the characteristic town policy from the perspective of sewage treatment in mountainous areas. Using micro-data, this study achieves results that cannot be obtained based on macro-data testing, expanding research in this area. It also provides a foundation for enhancing the comprehensive awareness of the effects of the characteristic town policy. Based on the preliminary findings and the features of characteristic towns, a series of robustness tests are conducted to assess the reliability of the study's results. Second, the theoretical mechanism of the impact of the characteristic town policy on mountainous sewage treatment is investigated, and the existence of investment and human resource effects is empirically verified. To further explore the complexity of the impact of the characteristic town policy on mountainous sewage treatment, this study tests the heterogeneity of the policy's impact in terms of economic development within different geographical regions. The study's findings will help theoretically expand the recognition of the socioeconomic effects of the Chinese government's urbanization policy.

## 2 Literature review

### 2.1 Status and treatment of urban sewage in China

Urban domestic and industrial wastewater is the primary source of pollutants in China's urban water resources. Urban sewage treatment has shifted from energy-consuming technologies based on pollution removal to efficiency-based technologies based on pollution removal and energy saving and the perspective to use capacity-based technologies based on synthetic utilization in the future [5]. China's urban sewage treatment capacity has gradually increased; however, the foundation of sewage treatment requires improvement. The sewage treatment capacity of most cities varies significantly depending on the requirements. Many sewage treatment plants operate under high loads and low discharge standards [6,7]. Deficiencies in research funding, treatment technologies, and management have constrained the development of urban sewage treatment technologies [8,9]. Urban sewage treatment programs have attracted the attention of several researchers. The improvement of urban sewage in China requires the vigorous promotion of water resource protection policies, the progress of management mechanisms, increased financial investment in urban sewage treatment, and the introduction of advanced foreign technologies [[10], [11], [12]]. Public-private partnerships have been widely adopted in sewage treatment, and the operational models and features of national demonstrations play a crucial role in reducing the volume of urban sewage treatment, supporting efficient and cross-regional sewage treatment [13]. The development of intelligent detection and artificial intelligence technologies has significantly improved the automation and intelligence of optimization setting technologies for future urban sewage treatment processes [14,15]. This trend has significantly improved the personnel's “intelligent” operation capability [16].

### 2.2 Current situation of rural sewage in China

One source of rural sewage is industrial pollution; however, the dominant sources are domestic, agricultural production, and agricultural farming sewage [17]. The characteristics of sewage discharge vary across areas. Domestic and livestock-breeding sewage are the primary sewage sources in the reservoir and water-source protection areas. In addition, sewage discharge patterns are influenced by the water habits of the villages. Villages in river network areas severely pollute the surrounding rivers, becoming an “invisible” pollution source. Rural sewage discharge from scenic tourist areas has prominent seasonal characteristics [18]. However, rural sewage is generally characterized by high water quality and quantity volatility, high organic content, and arbitrary discharge [19,20]. In addition, rural sewage status varies greatly owing to the distribution area, natural conditions, economic development level, scientific and technological power that could be invested, and living and production methods [21,22]. The proportion of rural sewage treatment in China's eastern, central, and western regions differs widely; in the eastern region, it is significantly higher than in the central and western regions. The rural domestic sewage in Jiaxing, a city in Zhejiang Province, shows decreases from west to east and north to south, declining in winter, spring, autumn, and summer sequentially over time [23,24]. In addition, large generation and low treatment ratio of domestic sewage is observed in rural areas of China. However, most rural areas lack sewage treatment facilities, and treatment efficiency is unsatisfactory, failing to meet the need to improve the quality of the rural living environment and promote the construction of beautiful and livable villages [[25], [26], [27]].

### 2.3 Rural sewage treatment in China

Compared to developed countries, China has not yet achieved a sound policy, legal system, and standard treatment system for rural domestic sewage treatment [28,29]. Issues such as the lack of awareness among rural residents regarding sewage treatment and perfect facilities in some areas, the low efficiency of sewage treatment, and the significant consumption of freshwater resources limit the quality of people's production and life [17,30,31]. Given the unpromising status quo of rural sewage treatment, scholars have conducted comprehensive studies on effective sewage treatment in such areas. Rural sewage comprises four main categories: centralized treatment - standard discharge, centralized treatment - resource utilization, decentralized treatment - standard discharge, and decentralized treatment - local utilization. The best choice for treating rural domestic sewage is local and nearby resource utilization, and reuse in agricultural production is the best way to utilize resources. However, owing to the lack of utilization standards and imperfect treatment systems, the resource utilization rate of rural domestic wastewater is relatively low. Therefore, a “resource utilization model for pollution and carbon reduction” should be developed to promote the effective development of rural wastewater treatment. Sewage-treatment technologies have also received considerable attention from researchers. With the help of Internet and new technology like big data, the bio-ecological combination treatment technology could meet the differentiated needs of rural domestic sewage treatment in China and had great potential for development and utilization [1,32,33].

Relevant studies have provided valuable references on this topic; however, further studies are required. First, existing studies have mainly analyzed rural and urban sewage treatment in China based on the treatment status quo and measures. However, policy-driven research on the effects of sewage treatment is needed, as well as analyses of the impact of national policies on improving urban ecological environments. Second, existing studies have focused on a unified research approach and summary of the problems and measures of rural and urban sewage treatment in China. Hence, the analysis of sewage treatment paths in different regions and urban clusters in China must be improved. Third, existing studies have primarily focused on the general background of rural revitalization policies for rural sewage treatment. Only a few studies have considered the characteristic town policy from a research perspective, and limited research has addressed the effect of characteristic town policy and sewage treatment in mountainous areas.

## 3 Theoretical mechanism

Given the macroscopic properties, public goods attributes, and externalities of natural resources and environmental issues, governments involved in the construction of small towns tend to invest limited resources, such as capital and labor, in public goods with high visibility, such as domestic sewage treatment, but fail to invest in goods with low visibility, such as underground sewage treatment facilities [34]. Therefore, small towns require assistance with sewage treatment due to their limited resources and acquired advantages. The widespread construction of characteristic towns has benefited the environmental management of small towns in terms of policies. As a result, local officials focus on implementing and promoting relevant policies under promotional incentives for environmental performance [35]. However, officials should further strengthen the construction of sewage treatment infrastructure and the breadth of coverage, enhancing the supervision and punishment of sewage discharges in rural areas and enterprises. In addition, the relevant laws and regulations exert specific deterrent effects on the potential risks of sewage treatment. Simultaneously, the intense supervision of characteristic town construction by the higher government promotes sewage treatment by regulating the behavior of the local government and encouraging government departments and officials to fulfill their responsibilities [36]. Finally, sewage treatment emphasizes the rational utilization of resources and strict restrictions on pollutant discharge. Therefore, considering the potential “market failure,” an “effective government” should strengthen sewage treatment policies to stabilize the expectations of relevant market entities and ensure the reasonable operation of an “effective market.” Hence, the first research hypothesis reads as follows.

H1 The characteristic town policy improves sewage treatment in small towns.

This article will elaborate on the impact mechanism of the characteristic town policy on sewage treatment from two perspectives: driving government and social investment and gathering human resources.

Driving government and social investment. The government's financial investment provides substantial financial support for constructing characteristic towns. As a powerful tool for the government's macroeconomic regulation, finance can effectively correct the adverse external effects of economic activities on the ecological environment caused by the “tragedy of the commons,” while compensating for the private sector's insufficient investment in environmental governance. Small towns on the cultivation list can receive adequate financial allocations from the central and local superior governments through financial rebates, bonus subsidies, and special fund subsidies. As a result, they have enough resources for environmental pollution management projects with a strong public welfare effect and a long investment return cycle, such as sewage treatment. Central financial incentive grants for small towns are politically more significant than the billions of funds invested in their construction. This result indicates that the central government considers a beautiful living environment an essential part of the construction of small towns. The central government's policy preferences may change the subjective beliefs of local governments regarding sewage treatment and amplify the impact on local government investment decisions through imitation effects. Moreover, although small towns can receive financial support from superior and local governments during construction, local governments rarely provide continuous and stable follow-up financial support due to increasing financial pressure in the long term. Hence, social capital investments must be introduced to maintain stable financing. Government investment plays a prominent role and drives social investment to support project financing in infrastructure, public services, and industry [37], such as the construction of sewage treatment infrastructure and the research and development of green sewage technology. Therefore, the characteristic town policy helps guide social capital investment through tax exemptions and environmental incentives and can have a powerful effect on enterprises, stimulating investment [38]. This phenomenon may induce environment-friendly enterprises to invest in and set up factories in towns. Moreover, it can limit the expansion of industrial enterprises to surrounding areas and alleviate their damage to the ecological environment within the region.

**Fig. 2:**
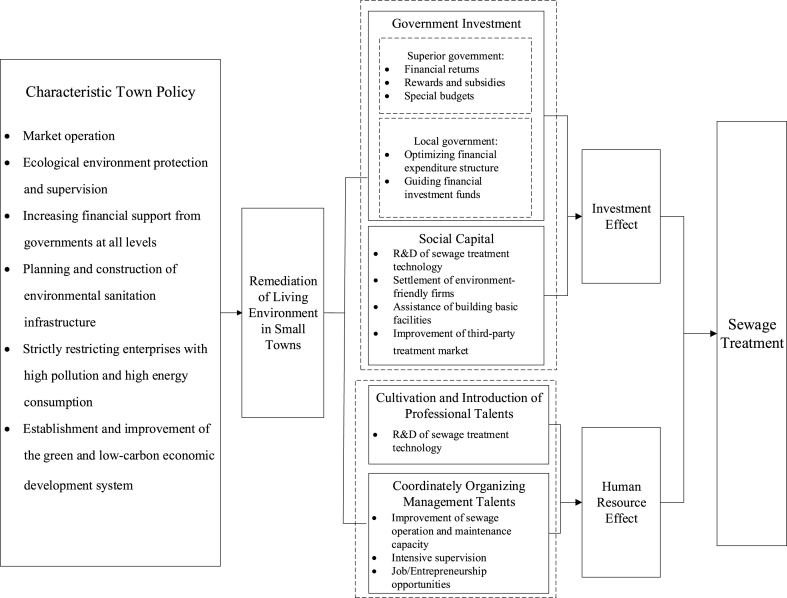
Theoretical mechanism of the effect of characteristic town policy on small towns.

Gathering human resources. The shortage of management personnel, especially professional personnel, inadequate regulation of sewage discharge, and insufficient operation and management in the later stages are significant drawbacks of sewage treatment in small towns [25] (Guo et al., 2022). However, due to their relatively poor economic development and geographical location, they may experience difficulties in attracting high-quality talent with technical and management experience to actively serve their construction. Investment in and formation of high-quality human capital in China are greatly influenced by policies and mainly relies on public finance. The characteristic town policy enables selected towns to receive substantial financial support from central and higher-level governments, which, in the long run, helps them cultivate environmental governance talent, such as the research and development (R&D) for sewage treatment technologies. In the short term, local and higher-level governments may help increase education and talent preferences to support characteristic towns by introducing and cultivating high-quality talent, management talent, and innovation teams. Such actions promote the development of innovative pollution control technology and improve the sewage operation and maintenance capabilities of frontline management personnel, quickly narrowing the talent gap in the town. Simultaneously, the superior government provides management personnel to assist the local government in sewage treatment, monitoring indiscriminate sewage discharge, and operating and maintaining the sewage treatment infrastructure. Finally, the construction of characteristic towns often accompanies regional economic restructuring, changes in industrial policies, and deepening transportation convenience, which promotes local employment and entrepreneurship opportunities and attracts relevant talent for active settlement. Hence, the second research hypothesis reads as follows (See Fig. 2.).

H2 The characteristic town policy improves sewage treatment in small towns by releasing investment and human resource effects.

## 4 Research design

### 4.1 Research area

Chongqing, in the upper reaches of the Yangtze River, has a landscape dominated by mountains and hills. It covers an area of 82,400 square kilometers, with 26 districts, eight counties, and four autonomous counties under its jurisdiction. Moreover, it combines large cities, rural areas, mountainous areas, and reservoirs. As of 2020, Chongqing had 778 small towns, of which 621 were towns and 157 were townships.^1^ This study chose Chongqing as the research objective for the following reasons. Chongqing is a representative region for sewage treatment in small towns located in mountainous regions. The proportion of mountains and hills in Chongqing is 98 %, of which only 2 % are river valleys and flat dams.^2^ In 2020, the total permanent population of Chongqing was 32 million, but 14.8505 million people lived in organic towns and townships, accounting for approximately 46.41 %.^3^ Therefore, enhancing sewage treatment in small towns is crucial to advance new urbanization with people at the core. Second, ecological and environmental protection in Chongqing is under pressure but is essential. Unlike other areas, Chongqing undertakes the vital task of building an ecological barrier in the upper reaches of the Yangtze River. It assumes an irreplaceable role in the ecological security of the middle and lower reaches of the Yangtze River. Approximately 80 % of the 19 districts and counties in the Three Gorges Reservoir are located in Chongqing.^4^ However, the municipality also faces various environmental pressures from a high proportion of its industries, scattered population in mountainous terrain, and rural environmental infrastructure that requires improvement.

In 2017, Chongqing selected 35 small towns from 35 districts and counties under its jurisdiction as characteristic towns to promote the successful development of industries in these towns and improve the ecological environment. Regarding policy support, the municipal government has granted 10 million yuan per year for five consecutive years of financial aid, and district and county governments have provided 1:1 matching funds. In addition, specific policies regarding land use and construction planning approval have been favored. The 35 characteristic towns in Chongqing are listed in Table 1.

**Table 1:** 35 characteristic towns of chongqing.

District and County	Small Town	District and County	Small Town	District and County	Small Town
Wanzhou	Wuling	Yongchuan	Zhutuo	Dianjiang	Gaoan
Qianjiang	Zhuoshui	Nanchuan	Dagan	Zhong	Shibao
Fuling	Linshi	Dazu	Longshui	Yunyang	Fengming
Shapingba	Qingmuguan	Qijiang	Dongxi	Fengjie	Baidi
Jiulongpo	Zouma	Tongliang	Anju	Wushan	Dachang
Beibei	Jindaoxia	Bishan	Fulu	Wuxi	Wenfeng
Yubei	Tongjing	Tongnan	Shuangjiang	Shizhu	Huangshui
Ba'nan	Dongwenquan	Rongchang	Wanling	Xiushan	Hongan
Wansheng	Qingnian	Kaizhou	Tieqiao	Youyang	Longtan
Changshou	Changshou Lake	Liangping	Pingjin	Pengshui	Baojia
Jiangjin	Baisha	Wulong	Jiangkou	Chengkou	Pingba
Hechuan	Laitan	Fengdu	Gaojia		

### 4.2 Model construction

#### 4.2.1 Differences-in-differences

The differences-in-differences (DID) method has been widely used in recent years to evaluate the effectiveness of policy implementation. This method views institutional change and the implementation of new policies as a “natural experiment” or “quasi experiment” external to the economic system [39,40]. It uses observational data to simulate an experimental research design, dividing the survey sample into two groups. One is the policy-affected group, the experimental group, and the other is the non-policy-affected group, the control group. First, this approach calculates the change in a specific indicator in the experimental group before and after the policy. Then, it calculates the change in the same indicator in the control group before and after the policy. Subsequently, the difference between these two variables is calculated to reflect the net impact of the policy. The DID method typically includes four elements: impact event, experimental group, control group, and period. The classical structure can be described as follows:

(1) $$Y_{it}=a+\delta D_{i}+\lambda T_{t}+\beta \left(D_{i}\times T_{t}\right)+\varepsilon_{it}\text{.}$$

In equation (1), $Y_{it}$ is the outcome variable. $D_{i}$ is a policy grouping dummy variable. $T_{t}$ is a policy time dummy variable. $D_{i}\times T$ is the interaction term, while δ, λ, and β are the coefficients of each term. $\varepsilon_{it}$ is a random error term. After obtaining the conditional expectation from the equation above, the estimated effect of the regression analysis can be obtained, where β represents the causal effect.

#### 4.2.2 Model design

Based on documents such as the *Opinions of the National Development and Reform Commission, the Ministry of Land and Resources, the Ministry of Environmental Protection, and the Ministry of Housing and Urban-Rural Development on Regulating the Promotion of Characteristic Towns and the Construction of Characteristic Towns*, provinces and cities have initiated policy formulations and the selection of characteristic towns since 2017. Implementing a pilot policy for characteristic towns in China may render sewage treatment in the same pilot town different before and after policy implementation. Moreover, this approach may cause sewage treatment to diverge between pilot and non-pilot towns. The model regression estimation based on this dual difference can effectively control for the effects of other synchronic policies and the ex-ante variation between pilot and non-pilot towns, thus identifying the net impact of policy shocks on sewage treatment in small towns. Therefore, China's small-town policy can be viewed as a “quasi-natural experiment,” and its effects can be evaluated using the DID approach. Based on the above discussion, this study first assesses the effectiveness of implementing the characteristic town policy on small-town sewage treatment using the following DID model:

(2) $$sewage_{it}=\alpha_{1}+\theta treated_{it}+\lambda controls_{it}+\eta_{i}+\mu_{t}+\varepsilon_{it}$$

The explained variable is the sewage treatment of small town i in year t, represented by the sewage treatment rate $rsewage_{it}$ and the total sewage treatment $tsewage_{it}$. The core explanatory variable $treated_{it}$ is a dummy variable reflecting whether small town i implements the characteristic town policy in year t. If small town i implements the policy in year t, the value is one, and zero otherwise $\eta_{i}$ and $\mu_{t}$ denote the fixed effects of small towns and years, respectively. $\varepsilon_{it}$ is a stochastic disturbance term affecting the sewage treatment rate of a small town. θ is an estimator of the DID effect, which measures the effectiveness of the characteristic town policy on the sewage treatment of small towns. $controls_{it}$ represents the control variables affecting the sewage treatment of small town i in year t. Referring to Chen and Zhang (2013), Jin et al. (2014), and Wang et al. (2019) [[41], [42], [43]], this study selects land urbanization, population urbanization, county radiation effect, and leading industry as control variables that influence the sewage treatment rate of small towns.

Land urbanization. Land urbanization is a direct manifestation of the expansion of urban areas. This phenomenon implies improving the essential public service facilities in small towns. This study adopts the ratio of built-up areas to the total area of small towns for measurement [44].

Population urbanization. Population is the core of urban development. Higher population urbanization implies greater agglomeration in small towns and less pressure on decentralized sewage treatment. Population urbanization is characterized by the resident population in built-up areas [45].

County radiation effect. Small towns are the “heads” of rural areas and the “tails” of cities: the economic and social development and construction of essential public service facilities are intimately associated with the magnitude of the county radiation effect [46]. The nearest distance between small towns and district and county governments is utilized as a measure in this study, with units of kilometers and data from Baidu Maps.

Leading industry. Small towns tend to have homogeneous leading industries due to their geographical location and population size [47,48]. According to the division of the Chongqing Housing and Urban-Rural Construction Commission, the leading industries of the 584 small towns in Chongqing comprise five categories: industry and mining, agriculture, business services, history and culture, and characteristic landscape. Since the economic development of small towns with a leading agricultural industry tends to be low, this study uses small towns whose top industry is agriculture as a reference group and the rest of the small towns as benchmark groups. It then examines the impact of leading non-agricultural industries on sewage treatment in small towns in Chongqing.

### 4.3 Sample selection and data source

We evaluate the impact of the characteristic town policy on mountainous sewage treatment based on Equation (2). To this end, this study chooses 2014–2020 as the research period and selects 584 organic towns in Chongqing as research samples, among which 35 small towns were characteristic. Among the four municipalities directly under the central government of China, Chongqing is the only one located in the west. Chongqing is a typical mountainous city, with mountainous areas accounting for over 75 % of its total area. Over 80 % of the world-renowned Three Gorges Reservoir Area is located in Chongqing, which exerts significant ecological pressure. Therefore, Chongqing is a representative area for studying mountainous sewage treatment in terms of the proportion of the mountainous regions and ecological pressure.

The core data for this study are obtained from the Chongqing Housing and Urban-Rural Construction Commission's Statistical Data on Urban and Rural Construction in Chongqing from 2015 to 2021. The Statistical Data on Urban and Rural Construction in Chongqing consist of three parts: cities, counties, villages, and small towns, with the part on villages and small towns covering organic towns, townships, and villages. According to the *China Urban-Rural Construction Statistical Yearbook 2021* of the Ministry of Housing and Urban-Rural Development of the PRC, as of 2020, there were 584 organic towns in Chongqing.^5^ Since this study has access to data from all 584 organic towns, no data screening or deletion is performed. According to the current administrative system in China, organic towns belong to the urban system, whereas non-organic towns and townships belong to the rural system. At the same time, considering the significant differences in economic development, urbanization, and population size between organic and non-organic towns and townships, this article only focuses on organic towns receiving substantial government investment to examine the effect of mountainous sewage treatment in characteristic towns. The results of descriptive statistics can be seen in Table 2.

**Table 2:** Descriptive statistics.

	(1)	(2)	(3)	(4)	(5)	(6)
VARIABLES	N	Unit	mean	sd	min	max
rsewage	4088	%	59.51	36.65	0	100
tsewage	4088	ten thousand cubic meters	20.17	35.75	0	680
lntsewage	4088	/	2.29	1.34	0	6.52
treated	4088	/	0.0323	0.177	0	1
lurban	4088	hectare	127.95	134.66	7.50	1750
purban	4088	ten thousand people	0.29	0.23	0	7.57
distance	4088	kilometer	39.12	22.23	3.40	146.30
invest	4088	ten thousand yuan	1599	5063	0	157,300
manager	4088	people	9.91	6.00	0	32
industry	4088	/	0.130	0.336	0	1

## 5 Results

### 5.1 Results of the benchmark model

The impact of the characteristic town policy on small-town sewage treatment is relatively complex, with multiple channels and forms. For comparison, columns 1 and 4 of Table 3 only include the core explanatory variables after controlling for town and year effects. The estimated coefficients of the dummy variable treated representing the characteristic town policy are 18.215 and 1.073, respectively, significantly positive at the 1 % level, indicating that the characteristic town policy remarkably enhances the sewage treatment status of the selected small towns.

**Table 3:** Benchmark regression.

	(1)	(2)	(3)	(4)	(5)	(6)
VARIABLES	rsewage	rsewage	rsewage	lntsewage	lntsewage	lntsewage
treated	18.215***	12.100***	11.305***	1.073***	0.740***	0.726***
	(5.11)	(4.65)	(4.77)	(7.71)	(8.98)	(6.32)
lnurbanarea		1.013***	1.163***		0.724***	1.848***
		(6.56)	(4.42)		(28.60)	(4.51)
urban		−9.338***	−4.193***		0.030	6.464***
		(-3.35)	(-1.73)		(0.33)	(2.92)
distance		−0.138***	−0.388***		−0.070***	−0.269***
		(-5.17)	(-3.37)		(-4.14)	(-5.08)
industry		0.671	0.653		15.204***	16.050***
		(0.39)	(1.29)		(6.24)	(3.02)
Town effect	Yes	NO	Yes	Yes	NO	Yes
Year effect	Yes	NO	Yes	Yes	NO	Yes
Constant	72.263***	44.582***	33.792***	4.012***	−0.760***	−91.200***
	(58.78)	(13.12)	(16.79)	(22.55)	(-7.01)	(-34.095)
Observations	4088	4088	4088	4088	4088	4088
R-squared	0.458	0.258	0.462	0.536	0.252	0.710

Note: Figure in the bracket is *t* value, and *p < 0.10、**p < 0.05、***p < 0.01. The same applies below.

Compared to ordinary small towns, sewage treatment in mountainous small towns faces difficulties caused by terrain, ecological environment, funding, and infrastructure. However, with the injection of large amounts of funds and policies from higher-level governments, the ecological environments of these towns have significantly improved. The implementation of the characteristic town policy has generated dividends for the selected small towns in terms of policy and investment. As discussed above, characteristic towns receive significant support from the superior government regarding industry selection, enterprise introduction, and town planning. Industries with heavy pollution and high energy consumption are restricted, while sewage treatment facilities receive significant financial support for construction and operation. For example, in 2020, the construction projects of 27 small towns among Chongqing's 35 characteristic towns involved constructing new and improving existing sewage treatment facilities, with a direct investment of more than 200 million yuan. Dongwenquan Town in Banan District restricted “small, scattered, disorderly” industrial enterprises to avoid severe damage to the ecological environment. Hence, Hypothesis H1 is confirmed, providing useful information for rural revitalization in China against the background of new urbanization.

To mitigate the effect of missing variables, four key factors, namely, land urbanization, population urbanization, county radiation effect, and leading industry, were controlled to obtain the results reported in Columns 2 and 5, respectively. The results demonstrate that the core estimated coefficients are reduced but statistically significant. Columns 3 and 6 control for town and year effects, respectively. The estimated coefficients of treated are 11.305 and 0.726, respectively, and their degrees of significance remain unchanged.

### 5.2 Parallel trend assumption

Benchmark analysis revealed the positive impact of the characteristic town policy on sewage treatment in small towns. However, this finding has an important underlying assumption: the treatment group should not differ significantly from the control group before the event. In this study, the selected small towns and others shared the same change trend in sewage treatment rate and total sewage treatment before policy implementation. The difference in trends in the previous period may bias the policy assessment if the reality violates the hypothesis. Therefore, this study redefines the sample time based on the first year of characteristic town policy implementation to verify whether the parallel trend hypothesis holds. We estimate the difference in sewage treatment between the selected small towns before and after the base year to establish the following model:

(3) $$sewage_{it}=\alpha_{1}+\sum \theta_{k}treated_{ik}+\lambda controls_{it}+\eta_{i}+\mu_{t}+\varepsilon_{it}\text{.}$$

**Fig. 3:**
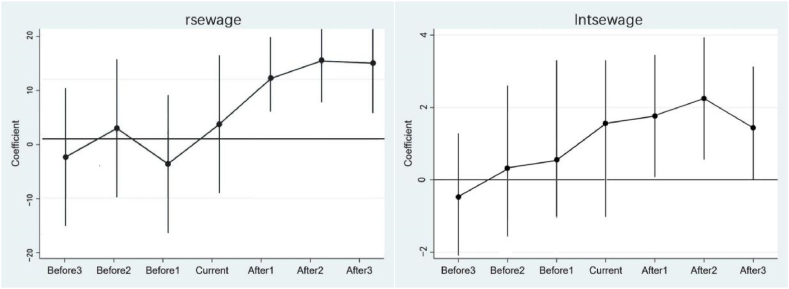
Parallel trend assumption. Note: Solid points represent the estimated coefficient $\theta_{k}$ in Equation (3), and the short vertical lines are the 95 % upper and lower confidence intervals corresponding to the robust standard error at the clustering level for small towns.

The variable $treated_{ik}$ is equal to one in the k-th year of the characteristic town policy, and zero in other years. This study selects the previous year of the implementation as the reference group. $\theta_{k}$ measures the differences in sewage treatment between characteristic towns and other towns in different years. If $\theta_{k}$ is statistically insignificant when k<−1, no difference is observed in sewage treatment between characteristic towns without the policy and other small towns, and the benchmark regression satisfies this assumption. $\theta_{k}$ measures the effect of sewage treatment in small towns implementing the policy when k≥0. If the coefficient is statistically significant, the characteristic town policy substantially impacts the sewage treatment model selected for small towns that year. Fig. 3 Plots the coefficients of $\theta_{k}$ in −3≤k≤3 and its 95 % confidence interval to directly observe the change in $\theta_{k}$. The results of the parallel trend assumption are reported in Fig. 3 And show the insignificance of the coefficients of different periods before the pilot policy of characteristic small towns. This result indicates no difference between characteristic towns and other towns before implementing the policy. The research sample respects the parallel trend assumption.

### 5.3 Placebo test

#### 5.3.1 Placebo test of time

This study conducts a time placebo test to avoid differences in sewage treatment between the small towns in the treatment and control groups caused by time variation. The implementation time of the characteristic town policy is advanced by one year, two years, and three years, respectively. This study constructs dummy policy times represented by $treated^{-1}$, $treated^{-2}$, and $treated^{-3}$. Regression analysis is conducted based on Equation (2), and the results are presented in Table 4. The results demonstrate that the estimated coefficients of $treated^{-1}$, $treated^{-2}$, and $treated^{-3}$ fail the significance test at the 10 % level. This result indicates no systematic difference in the time trend between small towns’ treatment and control groups, proving that the characteristic town policy improves sewage treatment.

**Table 4:** Placebo test of time.

	(1)	(2)	(3)	(4)	(5)	(6)
VARIABLES	rsewage	rsewage	rsewage	lntsewage	lntsewage	lntsewage
treated^−1^	3.25			0.56		
	(1.08)			(0.89)		
treated^−2^		4.13			0.47	
		(1.32)			(0.92)	
treated^−3^			6.20			0.67
			(1.39)			(1.08)
Control Variables	Yes	Yes	Yes	Yes	Yes	Yes
Town effect	Yes	Yes	Yes	Yes	Yes	Yes
Year effect	Yes	Yes	Yes	Yes	Yes	Yes
Constant	27.160***	29.345***	34.203***	−92.418***	−92.520***	−91.698***
	(7.69)	(13.12)	(17.88)	(-29.16)	(-31.83)	(-31.76)
Observations	4088	4088	4088	4088	4088	4088
R-squared	0.463	0.479	0.471	0.734	0.736	0.742

#### 5.3.2 Placebo test of small towns

**Fig. 4:**
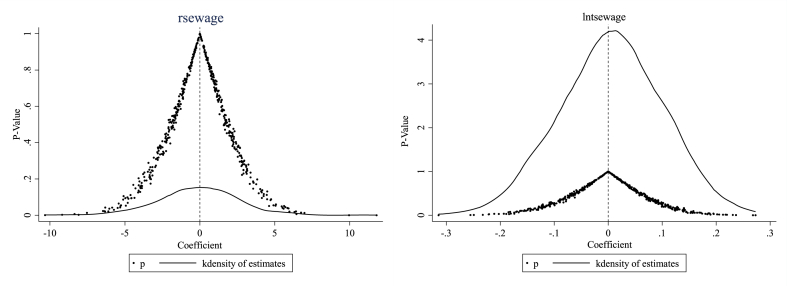
Coefficient distribution of placebo test of small towns (with *rsewage* and *lntsewage* as the explained variables).

To avoid the effects of unobservable missing variables on the benchmark regression results, a placebo test is conducted by substituting small towns in the treatment group, as described by Cai et al. (2016) [49]. In this study, 35 small towns are randomly selected from the sample as the dummy treatment group,^6^ and the remaining small towns are used as the dummy control group. Therefore, the estimated coefficients of the impact of implementing a small-town placebo for the characteristic town policy on small-town sewage treatment are obtained in Fig. 4. This process is repeated 500 times to obtain 500 regression coefficients and their corresponding p-values. The kernel density distribution and p-values of these 500 estimated coefficients are plotted to show that the regression coefficients fall near zero and follow a normal distribution. Most regression results are insignificant. Moreover, this finding indicates that the estimated values of the coefficients in the benchmark regression are located in the high tails of the distribution of spurious regression coefficients, which is a small-probability event in the small-town placebo test. Hence, it can be excluded that unobservable factors cause the benchmark estimation results in this study.

### 5.4 Robustness test

The benchmark regression results suggest that the characteristic town policy are conducive to improving sewage treatment in small towns. However, robustness tests are required to exclude confounding factors from the research findings. This study analyzes sample data filtering and excludes other policy interference to ensure the robustness of the estimated results.

(1) Sample data filtering. To avoid the impact of extreme values on the benchmark regression results, the research samples are trimmed by 1 % and 5 % according to the explained variables, and Equation (2) is re-estimated. The results in Table 5 indicate that, after excluding the extreme values, the estimated coefficients of the core explanatory variables are less different from the benchmark estimates in Table 3. All coefficients pass the significance test at the 1 % level. This result demonstrates that abnormal data do not affect the core findings of this study, and that implementing the characteristic town policy may improve the sewage treatment status in small towns.

**Table 5:** Robustness test.

	(1)	(2)	(3)	(4)	(5)	(6)
**VARIABLES**	rsewage	rsewage	lntsewage	lntsewage	rsewage	lntsewage
	Trimming 1 %	Trimming 5 %	Trimming 1 %	Trimming 5 %	Exclusion of other policy interference	
**treated**	17.101***	15.825***	0.230***	0.194**	15.360***	0.428
	(4.77)	(3.96)	(2.81)	(2.21)	(4.24)	(2.88)
**htown**					−0.144	−0.053
					(-1.21)	(-0.45)
**ttown**					0.469***	0.302***
					(2.65)	(2.70)
**Control Variables**	Yes	Yes	Yes	Yes	Yes	Yes
**Town effect**	Yes	Yes	Yes	Yes	Yes	Yes
**Year effect**	Yes	Yes	Yes	Yes	Yes	Yes
**Constant**	−13.792	−13.920	−1.450***	−1.476***	−13.521	−1.568
	(-0.24)	(-0.45)	(-13.47)	(-24.55)	(-0.22)	(-14.72)
**Observations**	4088	4088	4088	4088	4088	4088
**R-squared**	0.462	0.421	0.392	0.349	0.519	0.416
(2) Exclusion of other policy interferences. To control for the effect of different policies affecting small-town sewage treatment during the sample period, biasing the benchmark estimation results, this study identifies two policies that may significantly impact small: famous historical towns of China and national characteristic landscape tourism towns. Since 2003, famous historical towns in China have been jointly selected by the Ministry of Housing and Urban-Rural Development and the National Cultural Heritage Administration. As of 2020, 23 small towns in Chongqing have been chosen for this study. Since 2010, the Ministry of Housing and Urban-Rural Development and the former National Tourism Administration have jointly decided on national characteristic landscape tourism towns. As of 2020, 14 small towns in Chongqing have been listed. To avoid interference from other policies during the characteristic town policy, dummy variables for these policies are included in the benchmark regressions. htown is equal to one if a small town is a famous historical town in China that year, and zero otherwise. ttown is equal to one if a small town is a national-characteristic landscape tourism town that year, and zero otherwise. The results in Table 5 show that after excluding the two policies, the estimated effects for the core explanatory variables decrease slightly. However, the coefficients and significance are similar to those of the benchmark regression results, indicating that the main conclusions of this study are robust.

## 6 Further analysis

### 6.1 Impact mechanism test

According to previous theoretical analyses, a characteristic town policy may promote government investment in small-town sewage treatment by increasing government and social investment in construction and providing management personnel. This section attempts to test the possible mechanisms proposed above with econometric models, uses them as intermediate variables to directly examine the impact of the characteristic town policy on the intermediate variables, and constructs a mechanism verification equation. Furthermore, in terms of intermediate mechanisms to promote urban construction, numerous studies and empirical arguments have demonstrated the importance of financial investment [[50], [51], [52]] and personnel investment [53,54] for urban construction. Accordingly, this study builds the transmission chain of the characteristic town policy into an intermediate mechanism, thus improving small-town sewage treatment.

(1) Investment effect test. In line with previous theoretical analyses, we contend that the characteristic town policy may affect sewage treatment in small towns through increased urban construction investment. To verify this mechanism, the total urban construction investment, which includes government and social investment, served as a measure to ascertain the impact of the characteristic town policy on small-town sewage treatment through urban construction investment. Columns (1) and (2) of Table 6 present the estimated results of the impact of the characteristic town policy on sewage treatment. The results show that the estimated coefficients of treated are 0.278 and 0.288, which pass the significance test at the 10 % level. This result indicates that the characteristic town policy has increased urban construction investment under the combined policy and market effects. This study also tests the impact of urban construction investments on small-town sewage treatment. The results in Columns (3) and (4) of Table 6 reveal that the estimated coefficients of lninvest are 2.629 and 0.049, respectively, and both pass the significance test at the 1 % level. This result indicates that the increase in investment in urban construction has further contributed to improving the sewage treatment rate and growth in total sewage treatment. This approach is essential for the characteristic town policy to improve sewage treatment. The lack of infrastructure and operational difficulties are major challenges for sewage treatment in small mountainous towns. The increase in investment in urban construction can provide strong support for the construction of sewage treatment facilities in characteristic towns, such as sewage treatment plants, and offer financial support for the operation and maintenance of related infrastructure [55].

**Table 6:** The characteristic town policy affects the sewage treatment of small towns through urban construction investment.

	(1)	(2)	(3)	(4)
VARIABLES	lninvest	lninvest	rsewage	lntsewage
treated	0.278*	0.288*		
	(1.78)	(1.86)		
lninvest			2.629***	0.049***
			(7.67)	(5.36)
Control Variables	No	Yes	Yes	Yes
Town effect	Yes	Yes	Yes	Yes
Year effect	Yes	Yes	Yes	Yes
Constant	6.572***	7.707***	11.991	−1.746***
	(3.53)	(22.50)	(0.21)	(-15.64)
Observations	4088	4088	4088	4088
R-squared	0.503	0.552	0.570	0.719

(2) Human resource effect test. The implementation of the characteristic town policy may prompt a superior government to attach greater importance to small towns. In addition, supporting management personnel may be added along with increased capital investment. Simultaneously, sewage treatment is the focus of, and difficulty in, the construction of small towns. Local governments in small towns may increase the size of the management personnel, helping control indiscriminate rural discharge. To test the potential mechanism underlying the impact of supporting management personnel, we assess whether the characteristic town policy affects small-town sewage treatment by increasing the size of the supporting management personnel. This study uses the special management personnel of characteristic towns in the *Chongqing Urban-Rural Construction Statistical Yearbook* as a measurement index. Columns (1) and (2) of Table 7 report the estimated results of the impact of the characteristic town policy on supporting management personnel. The estimated coefficients of treated are 0.028 and 0.027, respectively, passing the significance test at the 5 % level. This result indicates that the characteristic town policy contributes significantly to the increase in supporting management personnel. This study also estimates the impact of supporting management personnel on sewage treatment in small towns. The results in columns (3) and (4) of Table 7 demonstrate that the estimated coefficients of lnmanager are 0.608 and 0.034, respectively, passing the significance test at the 5 % level. This result suggests that the characteristic town policy improves sewage treatment in small towns by increasing supporting management personnel. Therefore, Hypothesis 2 is supported. In China's current urbanization system, small towns are at the bottom, with relatively few resources available. In addition, small towns cover a wide area with numerous pollution sources and a shortage of personnel, making it difficult to restrict sewage treatment. Due to terrain factors, this problem is more prominent in small mountainous towns. Based on the above impact mechanism, it can be concluded that investment in urban construction and human resource improvement significantly impact sewage treatment in small mountainous towns, providing a theoretical foundation for China's green industry and environmental governance in rural revitalization.

**Table 7:** The characteristic town policy affects the sewage treatment of small towns through supporting management personnel.

	(1)	(2)	(3)	(4)
VARIABLES	lnmanager	lnmanager	rsewage	lntsewage
treated	0.028**	0.027**		
	(2.43)	(2.26)		
lnmanager			0.608*	0.034**
			(1.92)	(2.23)
Control Variables	No	Yes	Yes	Yes
Town effect	Yes	Yes	Yes	Yes
Year effect	Yes	Yes	Yes	Yes
Constant	0.668	2.092***	13.204	−1.581***
	(0.49)	(7.30)	(0.23)	(-13.96)
Observations	4088	4088	4088	4088
R-squared	0.239	0.369	0.492	0.616

### 6.2 Heterogeneity analysis

Previous studies have estimated the impact of the characteristic town policy on sewage management in mountainous areas. However, the heterogeneity of this impact will be further explored in this study, as different districts and counties differ significantly in their economic development and geographic locations, as well as in the difficulty of sewage treatment and the government's attention to sewage treatment.

#### 6.2.1 Different effects of the characteristic town policy on sewage treatment in small towns at different economic development levels

Based on the GDP per capita of 35 districts and counties from 2014 to 2020, this study classifies 12 districts and counties with GDP per capita reaching 64,792 yuan as high-level, 12 districts and counties with 45,355–62,738 yuan as medium-level, and 11 districts and counties with 23,223–42,347 yuan as low-level. This study uses Equation (2) to regress the sample data at different levels.^7^ Columns (1)–(3) of Table 8 present the estimated results of the effect of the characteristic town policy on the industrial impact of the sewage treatment rate in small towns with different economic development levels. In the high-level group, the estimated coefficient of treated is 28.268, passing the significance test at the 1 % level. Moreover, in the medium-level group, the estimated coefficient of treated is 15.128, passing the 5 % significance test. Finally, in the low-level group, the estimated coefficient of treated is 7.900, which passes the 5 % significance test. This result indicates a difference in the impact of characteristic town policy on sewage treatment in small towns with varying levels of economic development. Governments and enterprises should invest more funds in districts and counties with better levels of economic development to promote sewage treatment in characteristic towns. Therefore, the characteristic town policy strongly affects mountainous sewage treatment. In districts and counties with lower levels of economic development, the resources invested in constructing characteristic towns are relatively scarce because of limited financial income and social resources.

**Table 8:** Impact of characteristic town policy on sewage treatment in small towns with different economic development levels.

	(1)	(2)	(3)	(4)	(5)	(6)
VARIABLES	rsewage	rsewage	rsewage	lntsewage	lntsewage	lntsewage
	high-level	medium-level	low-level	high-level	medium-level	low-level
treated	28.268***	15.128**	7.900**	1.781**	0.355***	0.339**
	(4.28)	(2.39)	(2.36)	(2.45)	(2.66)	(2.37)
Control Variables	Yes	Yes	Yes	Yes	Yes	Yes
Town effect	Yes	Yes	Yes	Yes	Yes	Yes
Year effect	Yes	Yes	Yes	Yes	Yes	Yes
Constant	192.718***	−11.274	−16.413	−1.436***	−1.686***	−1.657***
	(2.95)	(-0.34)	(-0.11)	(-8.41)	(-9.50)	(-8.98)
Observations	805	1611	1672	805	1611	1672
R-squared	0.484	0.439	0.385	0.725	0.633	0.632

#### 6.2.2 Different effects of the characteristic town policy on sewage treatment in small towns in different regions

As the Three Gorges Reservoir Area is a significant ecological barrier in the upper reaches of the Yangtze River, the central and Chongqing municipal governments have devoted close attention to and given a certain degree of policy and financial preference for its green development. As previously mentioned, Chongqing covers over 80 % of the Three Gorges Reservoir. To examine the differences in the impact of the characteristic town policy on small-town sewage management in this area and other areas, this study divides the entire sample into 15 districts and counties in the Three Gorges Reservoir and the other regions for regression. Table 9 presents the impact of the characteristic town on small-town sewage treatment. In the Three Gorges Reservoir Area group, the estimated coefficients of treated are 36.177 and 2.113, respectively, both passing the significance test at the 1 % level. In other areas, the estimated coefficients of treated are 16.810 and 0.625, both passing the significance test at the 5 % level. This result suggests a significant difference between the effect of the characteristic town policy on sewage treatment in the Three Gorges Reservoir and that on small towns in other areas. Owing to the particularity of the Three Gorges Reservoir area, the characteristic town policy has substantially enhanced the sewage treatment of small towns in this area. Since 2015, the Chinese central government has focused on the ecological environment, with many resources invested in fragile ecological environments such as the Three Gorges Reservoir Area. In addition, the Three Gorges Reservoir is a mountainous area with relatively high sewage treatment pressure. As a result, compared to other small towns, the characteristic small towns that benefit from policy support have better sewage treatment results.

**Table 9:** Impact of characteristic town policy on sewage treatment of small towns in different areas.

	(1)	(2)	(3)	(4)
VARIABLES	rsewage	rsewage	lntsewage	lntsewage
	Three Gorges Reservoir area	other areas	Three Gorges Reservoir area	other areas
treated	36.177***	16.810**	2.113***	0.625**
	(6.45)	(2.19)	(2.69)	(2.36)
Control Variable	Yes	Yes	Yes	Yes
Town effect	Yes	Yes	Yes	Yes
Year effect	Yes	Yes	Yes	Yes
Constant	173.068***	−1.425***	−1.421***	−1.396***
	(3.10)	(-5.26)	(-12.87)	(-10.58)
Observations	1911	2177	1911	2177
R-squared	0.452	0.412	0.702	0.640

## 7 Conclusion and implication

### 7.1 Conclusion

With the continuous and in-depth implementation of rural revitalization strategies, the construction of small towns, especially characteristic towns, has become an essential part of rural revitalization strategies in China. Sewage treatment is an indispensable foundation for small towns’ high-quality economic and social development. However, this is a challenging task in urban construction. Mountainous and hilly areas in China are widely distributed, accounting for 43 % of the total land area. Many small towns within mountainous areas exhibit relatively low economic development levels, and prominent environmental infrastructure shortcomings face long-term environmental pressures caused by sewage discharge. Hence, this article uses data from 584 organic towns in Chongqing, a municipality directly under the central government in western China, known as a “mountain city,” to empirically analyze the effect and impact mechanism of the characteristic town policy implemented in 2017 on the sewage treatment of small mountainous towns.

This research found that the characteristic town policy helped improve sewage treatment in small towns. The conclusion remained valid after sample data filtering and excluding other policies’ interferences. The results of the mechanism test demonstrated that the investment and human resources effects were intermediate transmission mechanisms for the boosting effect of the characteristic town policy on mountainous sewage treatment. Further analyses have shown that this effect was heterogeneous regarding economic development and geographic location.

### 7.2 Implication

First, China should continue to promote the characteristic town policy in depth and fully release the policy dividends. The strength and breadth of the selection of characteristic national towns should be increased to fully play the role of an exemplary model. Superior governments should strengthen the administrative support for characteristic towns regarding industry cultivation, enterprise introduction, and regional planning. Local governments should introduce high-standard, strictly-specified and strongly-targeted sewage treatment regulations tailored to town characteristics. In addition, local governments should increase the supervision of sewage discharge from heavy-polluting and high-energy-consuming industries and support the development and growth of environmentally friendly enterprises, such as medical care, education, and the Internet.

Second, fully release the positive effect of investment and human resources on characteristic town policy to enhance sewage treatment in small towns. Government financial support should be strengthened through awards and subsidies, project subsidies, and tax exemptions to alleviate the financial pressure on polluting enterprises to reduce sewage. The government should also boost the construction and repair of rural sewage treatment infrastructure and allocate special funds to small towns for sewage treatment. In addition, we suggest actively developing financing channels for small-town sewage treatment and innovating enterprise financing products and models, particularly to provide green financial support. The government should be an excellent intermediary in guiding social capital investments. However, the government should also improve the allocation standards of sewage treatment management personnel in small towns, make a clear division of responsibilities among the personnel, and increase the number of management personnel in small towns where sewage treatment is relatively challenging. Discharges are more difficult to supervise if rural and corporate sewage discharges are around the clock.

Third, the policy should adapt to local conditions and focus on improving the economic development of small towns. When formulating and improving policies for characteristic towns, priority should be given to small towns’ economic development levels and geographical locations. For small towns with low levels of economic development, sufficient financial support should first be provided, followed by policy management support. Compared to small towns with less environmental pressure, those with more difficulties in sewage treatment should be given priority for government support to continuously improve the sewage treatment system of these towns in terms of policies, funds, and management personnel.

To sum up, this article utilizes differences-in-differences to explore the impact of Chinese the characteristic town policy on mountainous sewage treatment. With investment and human resource effects as mediators, a theoretical framework is preliminarily established for analyzing their relationship and relevant hypotheses are empirically tested, which helps theoretically expand the understanding of the impact of Chinese government's urbanization policies on the society and economy. At the same time, it is of great significance to effectively leverage the positive effect of China's characteristic town policy, improve the sewage treatment efficiency in mountainous small towns, and achieve high-quality development in the dual context of rural revitalization and green development. It should be pointed out that this study can benefit more from the data of all small towns across China. However, this article has not been able to obtain relevant data due to its availability, so further research is needed.

## Funding

This work was supported by the 10.13039/501100007957Technological Innovation Project of the Chongqing Education Commission [grant numbers KJCX2020040]; the Research Project of Sichuan International Studies University [grant numbers yjsjg202313]; the Research Project of Sichuan International Studies University [grant numbers SISUXWK202307].

## Data availability statement

Data will be made available on request.

## CRediT authorship contribution statement

**Chao Zhou:** Writing - original draft, Software, Methodology, Formal analysis, Conceptualization. **Qin Wang:** Writing - review & editing, Writing - original draft, Methodology.

## Declaration of competing interest

The authors declare that they have no known competing financial interests or personal relationships that could have appeared to influence the work reported in this paper.

## Footnotes

1 Data source: Chongqing Municipal Bureau of Statistics, Chongqing Statistical Yearbook 2022.

2 Data source: Chongqing Municipal Bureau of Statistics, Chongqing Statistical Yearbook 2022.

3 Data source: Chongqing Housing and Urban-Rural Construction Commission, Chongqing Urban-Rural Construction Statistical Yearbook 2021.

4 Data source: National Counterpart Cooperation Plan for the Three Gorges Reservoir Area (2021–2025) issued by the National Development and Reform Commission of the Ministry of Water Resources. http://www.gov.cn/xinwen/2021-12/20/content_5662885.htm.

5 Data source: Ministry of Housing and Urban-Rural Development of the People's Republic of China, *China Urban-Rural Construction Statistical Yearbook 2021*.

6 In 2017, Chongqing identified 35 characteristic towns. In order to maintain the same number, 35 small towns were also selected in the placebo test.

7 High-level districts and counties: Jiulongpo, Yubei, Fuling, Nan'an, Shapingba, Changshou, Bishan, Rongchang, Yongchuan, Ba'nan, Tongliang, Beibei. Medium-level districts and counties: Dazu, Jiangjin, Dadukou, Wanzhou, Liangping, Tongnan, Hechuan, Qijiang, Wulong, Dianjiang, Qianjiang, Nanchuan. Low-level districts and counties: Zhong, Xiushan, Shizhu, Fengdu, Kaizhou, Fengjie, Pengshui, Yunyang, Wushan, Wuxi, Youyang, Chengkou. The data source of GDP per capita is *Chongqing Statistical Yearboo*k from 2015 to 2020.
